# Editorial: School based physical activity: Can it work?

**DOI:** 10.3389/fspor.2022.1107274

**Published:** 2023-01-06

**Authors:** John G. Morris, Laura A. Barrett, Stephen F. Burns, Trish Gorely

**Affiliations:** ^1^ Department of Sport Science, School of Science and Technology, Nottingham Trent University, Nottingham, United Kingdom; ^2^ School of Sport, Exercise and Health Sciences, Loughborough University, Loughborough, United Kingdom; ^3^ Physical Education and Sports Science, National Institute of Education, Nanyang Technological University, Singapore, Singapore; ^4^ Department of Nursing and Midwifery, University of the Highlands and Islands, Inverness, United Kingdom

**Keywords:** health, exercise, cognition, children, adolescents, teaching, wearable technologies, sprints

**Editorial on the Research Topic** School based physical activity: Can it work?

If a healthy body is indeed as important as a healthy mind, it seems incongruous in countries such as the United Kingdom that so little time is devoted to physical activities strenuous enough to elicit health benefits in the school day/school curriculum. This is not a criticism of teachers and those responsible for the education of our children and young people. Simply that organisations such as the Chief Medical Officers of the UK and the Department of Health & Social Care in the United States make clear the widespread physical, cognitive and emotional benefits of a physically active lifestyle for children and young people (Department of Health & Social Care 2019; U.S. Department of Health and Human Services 2018) and yet we seem to be failing to ensure our young population engage in sufficient physical activity to maintain and promote health and other benefits. Theoretically, at least, school would appear to be the ideal structure and environment through which to ensure almost all children and adolescents have the opportunity to achieve sufficient physical activity to ensure they gain the positive health and other responses associated with a physically active lifestyle. Again, theoretically one could ask if not at school, then where and when?

A number of small-scale studies conducted in schools have clearly shown the benefits of even small quantities of exercise/physical activity on various health outcomes, even in otherwise “healthy” children and adolescents (Smallcombe et al. 2021). Given the focus in many societies on academic preparation and achievement in school it has to be recognised that the typically very busy school day offers little opportunity to increase physical activity for health and well-being. However, as the papers in this special issue indicate physical activity interventions can successfully take place in schools and can elicit benefits.

In their study, Wort et al. demonstrated that teachers were happy to utilise the data from wearable technologies to inform their behaviours, school practices and school policies aimed at encouraging physical activity; be this at a whole-school level or focussed on specific physical activity inequalities. Williams et al. conducted a short intervention which examined the effects of 6 sessions of six-to-eight 10-s sprints on cardiometabolic health and cognition. While no differences in markers of cardiometabolic health were seen, working memory was enhanced and BDNF concentrations were increased following the exercise intervention; the latter plays a role in neuroplasticity and memory. Hartikainen et al. examined whether the design of the learning environment (conventional self-contained classrooms vs. more flexible, informal open learning spaces) influenced sedentary behaviour. The findings from their study suggested that classroom structure alone had little impact on time spent being sedentary. The authors suggested that the methods of teaching *per se* were probably more important influences. Nally et al. examined what children perceived to be the barriers and facilitators to them being physically active during the COVID-19 pandemic, both during school but also at home. The study suggested that in school the barriers were lack of space and access to equipment; while the children thought that being able to go outside, to have fun and enjoy themselves were key facilitators. The children also thought that active breaks and/or incorporating movement in the classroom were desirable. The authors noted that peers and family were important influencers of children's physical activity behaviour.

Despite the promising findings noted above it must be recognised that a number of recent research studies, which have sought to summarise the available evidence examining the efficacy of school-based interventions on physical activity and health, have found little to no evidence of utility (Neil-Sztramko et al. 2021; Love et al. 2019). This is sobering. However, clearly in many cases children and young people need to be more active. If this is a societal need then the school day, probably in some adjusted format, would seem to offer an obvious mechanism through which opportunity for almost all can be ensured. Our inability to provide strong evidence of the benefits of school-based interventions on physical activity in practice may well suggest some error in reasoning. It may also be related to the complexity of the challenge, which involves diverse issues such as: the pressure on schools and their staff from multiple sources; general measurement challenges; and issues relating to individual preferences and behaviour change. However, the apparent disconnect between theoretical argument, small-scale study evidence and big picture outcome demonstrates that our understanding of how to ensure that the majority of our children and young people enjoy and undertake sufficient activity opportunities for health is actually poor.

Clearly, there is much more to do. Given the consequences of an “unhealthy” body we need to move, figuratively and perhaps literally, quicker.

